# Variable selection methods for descriptive modeling

**DOI:** 10.1371/journal.pone.0321601

**Published:** 2025-06-02

**Authors:** A. D. V. Tharkeshi T. Dharmaratne, Alysha De Livera, Stelios Georgiou, Stella Stylianou

**Affiliations:** 1 School of Science, RMIT University, Melbourne, Victoria, Australia; 2 Engineering and Mathematical Sciences, La Trobe University, Bundoora, Victoria, Australia; Semnan University, IRAN, ISLAMIC REPUBLIC OF

## Abstract

Variable selection methods are widely used in observational studies. While many penalty-based statistical methods introduced in recent decades have primarily focused on prediction, classical statistical methods remain the standard approach in applied research and education. In this study, we evaluated the variable selection performance of several widely used classical and modern methods for descriptive modeling, using both simulated and real data. A novel aspect of our research is the incorporation of a statistical approach inspired by the supersaturated design-based factor screening method in an observational setting. The methods were evaluated based on Type I and Type II error rates, the average number of predictors selected, variable inclusion frequency, absolute bias, and root mean square error. The detailed results of these evaluations are presented, and the methods’ performance is discussed across various simulation scenarios and in application to real data.

## 1 Introduction

Observational studies are prevalent in epidemiological and medical research. In these studies, statistical regression modeling is often employed to assess the associations between covariates (predictors) and the outcome of interest (descriptive modeling). However, as the number of available predictors increases, the goal often shifts toward fitting parsimonious regression models that include only a few predictors, specifically those that best explain the outcome (significant predictors) [1]. Variable selection is a common approach used to identify these important predictors [1–3].

The application of automated, data-driven methods is a popular approach for variable selection in real datasets. Classical methods such as forward selection, backward elimination (BE), and stepwise regression [4, 5] use a test-based approach, incorporating criteria like the Akaike information criterion (AIC) [6], Bayesian information criterion (BIC) [7] or *p*-values. Despite being in use since the 1960s and remaining widely used [4], these methods have been criticized for certain deficiencies [8–10]. Among these, the backward elimination method is most commonly applied in contemporary studies [11–22], particularly in the presence of collinearity [23].

In recent decades, more modern variable selection methods have been introduced, among which, penalized regression based variable selection is a more popular approach. The methods developed under this approach are introduced mainly focusing on high-dimensional data for prediction. These methods penalized least squares regression estimates and thereby shrink the coefficients of less important predictors to zero. The least absolute shrinkage and selection operator (LASSO) [24] was the first regularization method introduced incorporating L1-penalized regression for selecting significant predictors. It is often used as a benchmark in simulation studies to compare the performance of new methods [11]. Several extensions of LASSO have also been developed, incorporating different penalty functions [25–27]. Other modern methods include model averaging [2], correlation-based [28], tree-based methods [29–31] and Bayesian model averaging [32]. Variable selection is crucial in both low- and high-dimensional settings, and thus a number of modern methods are frequently introduced and/or applied in such contexts, see for example [33–37].

In a novel direction, experimental designs have been applied to select significant predictors from large observational datasets [38]. Particularly, supersaturated design (SSD), a form of fractional factorial design, is used for factor screening when data are gathered from a small number of sampling units (*N*) with a large number of predictors (*M*, where N≤M). This design is cost-effective and has been applied in experimental studies. [39, 40] extended SSD-inspired approaches for variable selection in large databases. These methods reduce the dimension of the dataset before applying existing variable selection techniques in observational studies. The statistical methods incorporated to analyze SSDs in the experimental setting were broadly discussed in the review of [41]. Many of these methods incorporated either modified or extended classical [42–49], penalized [50–52] regression methods, correlation-based [53] or Bayesian methods [54]. However, interestingly, several other methods have been introduced following procedures different to these popular approaches such as confidence intervals (CIs) [55], singular value decomposition [56], entropy measures [57], control charts [58] and model averaging [59] approaches. While methods developed for variable selection in observational studies have been successfully applied to SSDs for factor screening, the reverse—using SSD-specific factor screening methods for analyzing observational data—remains rare or virtually unused.

Several comparative studies have assessed the performance of the existing variable selection methods in observational studies, using simulation and/or real data. Many of these studies, even those focusing on low-dimensional settings, include statistical methods commonly applied to high-dimensional data [60–62]. However, many analyses are limited in scope, focusing on only a small number of methods [63–65] or exclusively on modern techniques [61, 66]. Several other studies which proposed new methods often compared only one to three alternatives, which may favor the proposed method. Furthermore, the studies which have evaluated multiple methods, tend to focus on predictive performance [62, 67, 68], ignoring the properties of the regression coefficient estimates. In descriptive modeling, however, it is important not only to select the correct predictors but also to produce valid statistical inferences about their effects on the outcome.

This paper addresses a critical gap in the literature by conducting a comprehensive comparison of variable selection methods in observational studies, with a focus on descriptive modeling. In particular, it aims to bridge the gap in applying SSD-based factor screening methods to observational studies, especially those involving low-dimensional data, where making inferences after variable selection is of primary interest. To achieve this, we extend a statistical method originally introduced for factor screening in SSDs, which was limited to binary input factors. This method performs variable selection using an iterative confidence interval (CI) approach [55]. The novelty of this paper lies in the evaluation and comparison of several statistical methods, including classical and penalized regression techniques, alongside the modified SSD-based factor screening method—referred to as the SSD-based confidence interval (SSD-CI) method. We assess the ability of these methods to identify true predictors, eliminate non-predictors, and make valid inferences about the selected predictors. By addressing these challenges, this research contributes to advancing the robustness and reliability of variable selection processes in observational studies. Practitioners will benefit from clearer guidance on selecting appropriate methods for identifying significant predictors and making valid inferences, ultimately enhancing decision-making in applied fields such as epidemiology, social sciences, and econometrics.

For inference, we conducted simulation studies using linear regression to generate the vector of continuous outcomes based on a subset of known true predictors with known non-zero coefficients. All the predictors included in this study were of continuous form. Given that the predictors were numeric, we adapted the SSD-based statistical method to accommodate various forms of observational data, extending its applicability beyond binary input factors.

This simulation study also considered multiple sample sizes, as previous research has shown that sample size and events-per-variable (EPV) (the ratio of sample size to the number of candidate predictors) [69], can impact variable selection performance.

Finally, we examined the stability of variable selection of each candidate statistical method using resamples drawn from a publicly available real-life dataset. This paper is organised as follows. In Sect 2, we describe both classical, modern variable selection methods included in this study and the SSD-CI method in detail. In Sects 3 and 4 we present the simulation and real data studies respectively and provide details regarding evaluation measures. In Sect 5, we present the results, followed by a discussion in Sect 6, and finally some concluding remarks in Sect 7.

## 2 Materials and methods

A linear regression model fitted on an observational dataset which consists of *N* observations and *M* covariates with a continuous outcome is presented as

$$𝐘=\beta_{0}+𝐗\beta +\epsilon ,$$ (1)

where, **Y** is the N×1 vector of outcomes, $\beta_{0}$ is the intercept, **X** is the N×M model matrix with *x*_*ij*_ being the data collected from the *i*^*th*^ subject on the *j*^*th*^ predictor (where i=1,2,…,N and j=1,2,…,M), β ($=\beta_{1},\beta_{2},\ldots ,\beta_{M}$) is the M×1 vector of coefficients of the predictors and ϵ is the N×1 vector of residuals/error terms which is normally distributed with zero mean and covariance matrix $\sigma^{2}I_{N}$, with σ unknown. This model, known as the screening model, suggests that only a few of the predictors being investigated are significant, as posited in SSD’s methods (effect sparsity assumption).

We now present the variable selection methods incorporated in the comparative study to assess the performance of identifying the significant predictors along with making valid inferences. Under classical methods, the BE method, which is popularly used in biomedical researches is considered. Under modern variable selection methods, LASSO, Adaptive LASSO (ALASSO), Elastic Net (ENet), smoothly clipped absolute deviation (SCAD), minimax convex penalty (MCP) and iterative sure independence screening methods are considered. We also explained the process of selecting potentially significant predictors by the iterative confidence interval (CI) approach inspired by SSDs (SSD-CI) in an observational setting. We now explain the statistical procedure followed by each considered method in selecting the potentially significant predictors.

### Classical variable selection method: Backward elimination

Backward elimination (BE) [70] begins with the full model, including all predictors and then eliminates the least significant predictors step-by-step until all the predictors in the model become significant. This study assessed the significance of the predictors based on *p*-value approach with (p=0.05), AIC and BIC [71] criterion. The methods with the three selection criterion are therefore, denoted as BE(0.05), BE(AIC) and BE(BIC), respectively. In this study, variable selection based on AIC and BIC approaches was performed using the *step* function in the R software while selection based on *p*–value approach was made using the *olsrr* [72] package in the R software.

#### Modern variable selection methods.

Under modern variable selection approaches, seven popular penalized regression methods were considered. These methods estimate the regression coefficients as:

$$\hat{\beta }=\underset{\beta }{\mathrm{argmin}}\frac{1}{2N}||Y-X\beta |{|}_{2}^{2}+\lambda (pen(\beta ))\;$$ (2)

where pen(β) is the penalty term and its strength is controlled by the tuning parameter, λ(≥ 0). The most common approach of estimating λ is by using the cross-validation (CV) method [73]. Different forms of penalty functions utilized by the penalized methods considered in this study are discussed below:

**Least absolute shrinkage and selection operator.** Least absolute shrinkage and selection operator (LASSO) [24] imposes ${\text{L}}_{1}$ - norm penalty in Eq (2) as $\lambda (pen(\beta ))=\lambda ||\beta |{|}_{1}=\lambda \sum_{j=1}^{M}|\beta_{j}|$. In this study, LASSO was performed using the *glmnet* package [74] in the R software using two different values for λ: a) λ which produces minimum mean cross-validated error $\left(\lambda_{min}\right)$ and b) the largest value of λ that is within 1 standard error of the cross-validated errors for $\lambda_{min}$ $\left(\lambda_{1se}\right)$. The optimal values of $\lambda_{min}$ and $\lambda_{1se}$ were determined using 10-fold CV and the two versions of LASSO are labelled as LASSO$\left(\lambda_{min}\right)$ and LASSO$\left(\lambda_{1se}\right)$, respectively.

**Adaptive LASSO.** Adaptive LASSO (ALASSO) [26] is an extension of LASSO, which introduces weights to the penalty term of Eq (2) as $\lambda (pen(\beta ))=\lambda \sum_{j=1}^{M}w_{j}|\beta_{j}|$, based on which, the predictors are penalized with different magnitudes. In this study, each weight component was computed using the ridge regression coefficient estimate corresponding to $\beta_{j}$, as $w_{j}=1/|{\hat{\beta }}_{ridge,j}|$. Furthermore, similarly to LASSO, variable selection was performed incorporating both types of λ estimates. Therefore, we denote the methods under the two λ cases as $ALASSO(\lambda_{min})$ and $ALASSO(\lambda_{1se})$. ALASSO was conducted using the glmnet package [74] in the R software.

**Elastic Net.** Elastic Net (ENet) [25] introduces a penalty term combining ${\text{L}}_{1}-$ and ${\text{L}}_{2}-$ norm penalties with an additional parameter α∈[0,1] as $\lambda (pen(\beta ))=\lambda (\alpha ||\beta |{|}_{1}\;+\;\frac{\left(1-\alpha \right)}{2}||\beta |{|}_{2}^{2})=\lambda (\sum_{j=1}^{M}\alpha |\beta {|}_{j}\;+\;\frac{\left(1-\alpha \right)}{2}\beta_{j}^{2})$. The optimal values for the hyper-parameter λ and α were computed via 10-fold CV using the *glmnet* package [74] in the *caret* package platform [75] in R software.

**Smoothly clipped absolute deviation.** The Smoothly clipped absolute deviation (SCAD) [76] utilizes a non-convex penalty term to estimate the regression coefficients and perform variable selection as;


$$\lambda (pen(\beta_{j}))=\left\{ \begin{array}{cc} \lambda |\beta_{j}|, & |\beta_{j}|\leq \lambda \\ {}[8pt]\frac{2\gamma \lambda |\beta_{j}|-|\beta_{j}{|}^{2}-\lambda^{2}}{2(\alpha -1)}, & \lambda <|\beta_{j}|\leq \gamma \lambda \\ {}[8pt]\frac{(\gamma +1)\lambda^{2}}{2}, & |\beta_{j}|>\gamma \lambda \end{array}\right.$$

where γ>2 and λ>0. These non-linear penalties tend to select potentially significant variables by only shrinking the small coefficients towards zero. In this study, variable selection using SCAD was implemented using the *ncvreg* package [77] in R software. We set α to be 3.7 [78] as recommended in [76] and also the default value in the *ncvreg* package [77]. The optimal value of λ was determined using 10-fold CV to minimize the CV error.

**Minimax convex penalty.** The minimax convex penalty (MCP) [79] is another variable selection method which applies non-linear penalties in the regularized regression in the form of;


$$\lambda (pen(\beta_{j}))=\left\{ \begin{array}{cc} \lambda |\beta_{j}|-\frac{|\beta_{j}{|}^{2}}{2\gamma }, & |\beta_{j}|\leq \gamma \lambda \\ {}[8pt]\frac{1}{2}\gamma \lambda^{2}, & |\beta_{j}|>\gamma \lambda \end{array}\right.$$

for γ>1 and λ≥0. Similarly to SCAD, these non-linear penalties tend to apply less shrinkage on large coefficients compared to the small coefficients. In this study, MCP was implemented using the *ncvreg* package [77] in R software. We set γ to be 3 which is the default value in the *ncvreg* package [77]. The optimal value of λ was subsequently determined using 10-fold CV.

#### Iterative sure independence screening.

Iterative sure independence screening (ISIS) [28] is a two-stage approach that iteratively applies the Sure Independence Screening (SIS) method for predictor selection, as outlined below.

SIS is commonly used in high-dimensional settings where, *N*<*M* to reduce dimensionality by screening predictors from *M* down to a manageable size, d(<N). (For details on SIS and the computation of *d*, see [28]). In brief, predictor screening is made based on the N×1 vector ω computed using componentwise regression:


$$\omega =X_{std}^{T}Y,$$

where $X_{std}^{T}$ is the transpose of the standardized model matrix and Y is the response vector. The *M* components of ω are ranked in descending order, and the top *d* predictors with the highest absolute $\left|\omega_{j}\right|$ values are retained. The coefficients of these selected predictors are then estimated and those close to zero are eliminated using a penalized regression method such as SCAD or LASSO, resulting in *d*_1_ (≤d) selected predictors. In this study, we used the *SIS* package [80, 81] in R, where SCAD is the default penalization method, referring to this approach as SIS-SCAD.

ISIS, begins by constructing an initial submodel, *A*_1_, containing the *d*_1_ predictors selected via SIS-SCAD. The response Y is then regressed on these predictors, generating an *N*-vector of residuals. In the next step, treating this residual vector as a new response, SIS-SCAD is applied to the remaining $M\;-\;d_{1}$ predictors. This process identifies another subset of *d*_2_ predictors, forming submodel *A*_2_, and a new set of residuals is obtained. The procedure repeats iteratively until *D* disjoint submodels are formed. In high-dimensional settings, the size of the union of $A={⋃}_{j=1}^{D}A_{j}$, remains below *N*. Finally, SCAD is applied once more to the combined set of predictors in *A* to eliminate those that are insignificant in the presence of others.

#### Supersaturated design based statistical approach.

The design of SSD-based confidence interval method (SSD-CI method) was inspired by the two-stage contrast-based screening method proposed by [55] for factor screening in two-level SSDs (where each input factor consist of two levels) with a continuous outcome. The SSD-CI method selects the significant variables by constructing CIs in an iterative (*k*) process as explained below:

1. Compute the contrasts for each variable $\left(C=C_{1},C_{2},...,C_{M}\right)$ as: $C=X_{std}^{T}Y.$
2. Order the absolute contrasts as $\left|C_{\left(1\right)}|\leq |C_{\left(2\right)}|\leq \ldots \leq |C_{\left(M\right)}\right|$.
3. The iterative process (*k*) then begins. At the beginning of the *k*^*th*^ iteration, the available largest absolute contrast is removed. Then, the variance of the remaining “*m*” largest absolute contrasts are computed. In this study, the variance is computed by incorporating 75% of the variables available in the *k*^*th*^ iteration. The CI for the *k*^*th*^ iteration is then generated using the computed variance. Of note, this process begins by setting *k* to 0. Therefore, the initial CI (i.e. at *k* = 0) is computed incorporating all contrasts. For more details on computing the CIs, please refer [55] study. The generalized iterative process for the generation of CIs in each iteration is explained in detail below.
  Calculate the variance $\hat{\sigma_{m,k}^{2}}$ using the “*m*” largest absolute contrasts in the *k*^*th*^ iteration (i.e., the variance of $\left|C_{\left(M-k-m+1\right)}|,\ldots ,|C_{\left(M-k\right)}\right|$).
  Construct the critical region using the following upper and lower limits:
    $$ucl_{k}=|C_{M-k}|-t_{M-k-1,\alpha /2}{\hat{\sigma }}_{m,k}\text{ and }lcl_{k}=-|C_{M-k}|+t_{M-k-1,\alpha /2}{\hat{\sigma }}_{m,k},$$
  where, $t_{M-k-1,\alpha /2}$ is the critical value of the *t*-distribution with (*M*–*k*–1) degrees of freedom and α(=0.05) is the level of significance.
  Check if $\hat{\sigma_{m,k}^{2}}<\hat{\sigma_{m,(k-1)}^{2}}$ (of note, the variance of step *k*–1 was set to be ∞ at *k* = 0 case), $|C_{M-k}|>t_{M-k-1,\alpha /2}{\hat{\sigma }}_{m,k}$ and (M−(k+1))>m.
    If all three conditions satisfies, increase the iterative process by one (*k* = *k* + 1), remove the largest absolute contrast value |*C*_*M*−*k*_| from C and go to step 3.1.
    Otherwise terminate the iteration process. If one of the first two conditions are violated, then the variables are selected using the critical region generated in the (*k*−1)^*th*^ iteration [$\left(lcl_{k-1},ucl_{k-1}\right)$] and if the third condition is violated, variable selection is done using the critical region generated in the *k*^*th*^ iteration [$\left(lcl_{k},ucl_{k}\right)$]. The variables of the contrasts which fall outside the limits of the selected CI, are selected as potentially ‘significant’ variables.

The R code of the SSD-CI method has been provided and made freely available for academic use (Table B in S1 Appendix). Of note, the original method is a screening method, hence, limits to performing variable selection. Therefore, in this study, for inference purpose, following variable selection, a linear regression model was fitted with the data in the model matrix corresponding to the chosen variables and the original response. Then, the resulting regression coefficient estimates were stored as the coefficient estimates provided by this method.

## 3 Simulation study

We performed a simulation study to assess the variable selection performance and making valid inference of the aforementioned methods. The datasets were simulated with a fixed number of predictors where all of them are numeric but with different sample sizes (EPVs) as detailed below.

### 3.1 Data generation

The generation of the simulated data was inspired by the technical report of [82] and [18] study. The authors in those papers simulated data reflecting biomedical data comprising of both continuous and categorical factors. In this simulation study, however, datasets with numeric predictors and continuous outcome were considered. This study evaluated the variable selection performance of statistical methods under varying conditions, including different magnitudes of multicollinearity and true coefficient values, [18] to assess their ability to accurately identify true predictors.

In brief, the data generation process initiated with constructing a **Z** matrix by drawing 15 standard multivariate normal deviates with a pre-specified correlation structure (between -0.3 and 0.8) [18, 82]. These values were then transformed (*T*) to yield the data matrix 𝐗(=T(Z)) resulting 15 predictors ${𝐗}_{j},j=1,2,\ldots ,15$ with various marginal distributions. Details for the distributions of the predictors and the correlation structure are provided in Figs A and B in S1 Appendix, respectively. We then set the first 7 predictors to have a true influence on the outcome. Hence, for β, non-zero values were applied for the first seven predictors denoting “true predictors" and zero was assigned for the remaining (“false predictors"). Finally, the outcome **Y** was computed using Eq (1). Therefore, the “true model" involved in the simulation study is


$$𝐘=11.206+0.041X_{1}-1.061X_{2}+0.249X_{3}+0.625X_{4}\;\;+0.104X_{5}+0.022X_{6}-0.01X_{7}+\varepsilon ,$$

with $\varepsilon \sim N(0,0.868I_{N}).$ Additionally, ℓ=1000 datasets with sample sizes 225, 375, 500, 750 and 1000 were simulated. These sample sizes were specifically selected to represent the EPV regions under which variable selection is suggested to perform according to [22]. Therefore, these selected sample sizes represented the EPVs 15, 25, 33.3, 50 and 66.7, respectively. Each candidate statistical method was then applied on each dataset simulated under a specific sample size. The selected predictors were considered to be “significant" predictors under the given method and sample size. The coefficient estimates corresponding to the selected predictors were also stored to test for validity of inference.

### 3.2 Measures of performance in simulation study

Following variable selection, we compared the ability of each method on correctly identifying the true state of each predictor and making valid inferences on the selected regression coefficients using several summary measures. Firstly, model performance was evaluated using Type I error rate and Type II error rate. Selection stability was then assessed using variable inclusion frequency (VIF) and average number of predictors selected by each method. The VIF measure was computed for each predictor separately while the error rates and average number of predictors selected were computed involving all predictors. Additionally, absolute bias and root mean square error (RMSE) were computed for each predictor for inference perspective. The evaluation criterion of each summary measure is detailed below:

- **False positive rate and false negative rate.** False positive rate (FPR) and false negative rate (FNR) are also defined as Type I error rate and Type II error rate, respectively. In each simulation, Type I error is calculated as the number of falsely selected non-predictors divided by the number of true non-predictors; 8 in this setup. Similarly, Type II error is calculated as the fraction of the number of true predictors which were not selected as significant divided by the number of true predictors; 7 in this setup. These errors were then averaged over the total number of simulations (ℓ) to obtain FPR and FNR, respectively. The closer these values are to 0, the better is the performance.
- **Average number of predictors selected.** Following variable selection using a specific method in each simulation, the number of predictors selected as significant was recorded without considering the true state (true predictor or not) of the predictor. The number of predictors selected across all the simulations are summed up and then divided by the the total number of simulations (ℓ), to obtain the number of predictors likely to be selected by the specific method on average.
- **Variable inclusion frequency.** The variable inclusion frequency (VIF) investigates how often a candidate method selects each predictor as “significant" and thereby identify which of the “true" predictor(s) are less likely to be selected by the specific method. In order to compute the VIF for a specific predictor, the number of times the predictor was selected over the ℓ simulations is divided by the number of simulations (ℓ) and multiplied by 100 to present it as a percentage. It is expected to record values closer to 100% for the true predictors (i.e. the first 7 input factors) and values closer to 0% for the false predictors ($X_{8}\;-\;X_{15})$.
- **Absolute bias and root mean square error.** Absolute bias and root mean square error (RMSE) were computed for each regression coefficient by taking in to account of the regression coefficient estimates computed for each predictor in each simulation and the true regression coefficient values assigned for each predictor in the true model. Bias for a predictor was computed by summing up the differences between each coefficient estimate and the true coefficient value corresponding to the predictor over the simulations and then dividing it by the ℓ number of simulations. (Table 6 of [83]). Since we are not concerned with overestimates or underestimates, we focus solely on the magnitude of the bias. Therefore, we calculate the absolute bias to provide a clear representation. MSE for each regression coefficient was estimated by calculating the summation of squared differences between each coefficient estimate and the true coefficient over the ℓ simulations and then dividing that by the total number of simulations (ℓ). The RMSE is then yielded by taking the squareroot of the MSE (Table 6 of [83]). An absolute bias and RMSE magnitude close to 0 across all the predictors indicate a reliable method for inference.

## 4 Real data application

To evaluate the performance of the candidate methods in real-world applications, we applied them to a red wine dataset [84], available from the UC Irvine Machine Learning Repository https://archive.ics.uci.edu/ml/datasets/wine+quality, which contains data relevant to red and white variants of the “Vinho Verde” wine, from the north of Portugal.

The potential of hydrogen (pH) is a key indicator of wine quality, particularly, in red wine, where it influences colour intensity, taste, chemical stability and the effectiveness of sulfur dioxide. Using the red wine data, we identified the predictors most associated with pH levels through the candidate statistical methods. The analysis included only the ten numerical predictors, yielding approximately 160 events per variable (EPV = 1599/10).

### 4.1 Stability measures in real data application

In the application of a variable selection method on a real dataset, [22] recommended to investigate the stability of variable selection. For this purpose, instead of applying each method on the complete dataset, we drew resamples from the original dataset. Moreover, [18] study showed that VIF and model selection frequency (MSF) should be identified following subsamples while stability measures need to be estimated based on bootstrap samples. Therefore, to compute VIF and MSF for the real dataset, 1000 subsamples (*n*_*res*_) were drawn where for each subsample, 50% of the original dataset was randomly selected without replacement (M=⌊0.5N⌋). Due to the absence of knowledge about true predictors in real-life applications, the stability measures relative conditional bias (RCB) and root mean squared difference (RMSD) ratio were estimated instead of bias and RMSE by using 1000 bootstrap samples (*n*_*res*_) (each sample is drawn with replacement from the original dataset, maintaining the dataset’s size). Each candidate method was applied on both the original dataset and each resampled dataset and computation of each stability measure is explained below.

The VIF measure for each predictor was then computed similarly as explained under simulation study, but using subsamples, instead (sub_VIF). Moreover, the model that was most frequently selected following application of each specific method on the 1000 subsamples was considered and the frequency of it was recorded as the MSF (sub_MSF). Both RCB and RMSDR were computed as explained in [22].

In brief, RCB of a coefficient is calculated as:


$$\text{RCB}(\beta_{j})=\left[\frac{{\hat{\beta }}_{sub,j,l}}{{\hat{\beta }}_{global,j}\cdot \text{sub\_VIF}}-1\right]\times 100\%,$$

where ${\hat{\beta }}_{sub,j,l}$ is the subsample estimate computed for the *j*^*th*^ predictor under the *l*^*th*^ subsample $(l=1,\ldots ,n_{res}$), ${\hat{\beta }}_{sub,j,l}$ is the mean of the subsample estimates of the *j*^*th*^ predictor and ${\hat{\beta }}_{global,j}$ is the global model (multiple linear regression model (Eq (1)) fitted using complete dataset including all predictors) estimate corresponding to the *j*^*th*^ predictor.

RMSD of a coefficient is computed as:


$$\text{RMSD}(\beta_{j})=\sqrt{\frac{\sum_{l=1}^{n_{res}}({\hat{\beta }}_{sub,j,l}-{\hat{\beta }}_{global,j}{)}^{2}}{n_{res}}}.$$

RMSD ratio (RMSDR) is finally computed by dividing the RMSD by the standard error of the coefficient of the respective predictor in the global model.

## 5 Results

### 5.1 Performance evaluation based on simulation study

**Fig 1:**
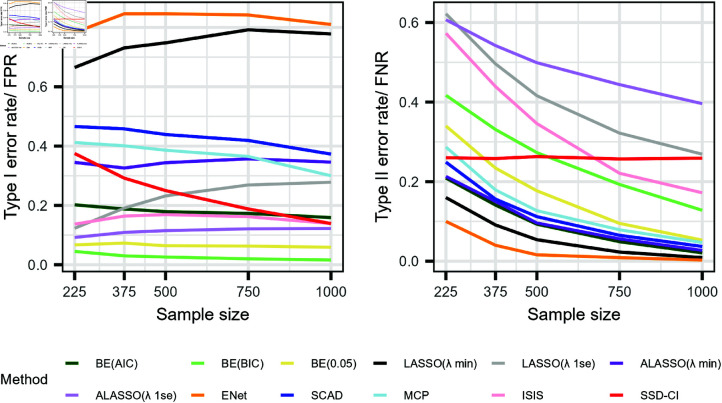
Overall summary statistics of the twelve variable selection methods with all simulated predictors. 1000 simulations were used to plot and evaluate the variable selection performance of twelve candidate methods with respect to (A) Type I error rates (lower values preferable) and (B) Type II error rates (lower values preferable) under different sample sizes (*N* = 225, *N* = 375, *N* = 500, *N* = 750) and *N* = 1000). twelve compared variable selection methods represented by coloured lines: BE(AIC), BE(BIC), BE(0.05), LASSO($\lambda_{min}$), LASSO($\lambda_{1se}$), ALASSO($\lambda_{min}$), ALASSO($\lambda_{1se}$), Elastic Net, SCAD, MCP, ISIS and SSD-CI.

Fig 1 presents the variable selection performance of the candidate methods with respect to the overall summary statistics (FPR and FNR) under five differing sample sizes. Elastic net included the largest number of false predictors by recording an FPR exceeding 0.75 (including at least 6 of the 8 false predictors on average) followed by LASSO($\lambda_{min}$) (FPR beyond 0.625 with at least 6 of the 8 of the false predictors selected on average), despite recording the lowest FNRs. BE(BIC) excelled in avoiding false predictors followed by BE(0.05) computing an FPR below 0.1 (Fig 1A). However, BE(AIC) and ALASSO($\lambda_{min}$) outperformed with respect to selecting the true predictors by only dropping at most 2 of the 7 true predictors on average (FNR: 0.209 and 0.213, respectively) at the small sample case and since *N* = 500, this rate was dropped below 0.1 denoting at most 1 of the true predictors will be eliminated from the final model on average. SCAD and MCP were the next best methods in capturing the true predictors by recording an FNR below 0.3 in all cases.

In contrast, ALASSO($\lambda_{1se})$ and LASSO$(\lambda_{1se}$) were the weakest in identifying the true predictors. At *N* = 225, they dropped at least 4 of the 7 true predictors (FNR just above 0.6) from the final model on average. However, the performance of capturing true predictors improved with the sample size by dropping the FNR to 0.396 and 0.269, respectively at *N* = 1000 by only dropping 3 and 2 true predictors, respectively. Although, LASSO($\lambda_{1se}$) had a higher FNR compared to SSD-CI method throughout the study samples, it is expected to drop below SSD-CI method beyond *N* = 1000. The main reason is that the FNR of the SSD-CI method remained constant at about 0.25 regardless of sample size, while other methods showed improvements. However, LASSO($\lambda_{1se}$) struggled with false predictor inclusion as the sample size increased, which allowed ISIS to outperform it in both error rates across all sample sizes except at *N* = 225. Interestingly, the SSD-CI method recorded the highest rate in omitting false predictors resulting in recording an FPR of 0.259 at *N* = 1000-lower than BE(AIC) and comparable to ISIS.

**Fig 2:**
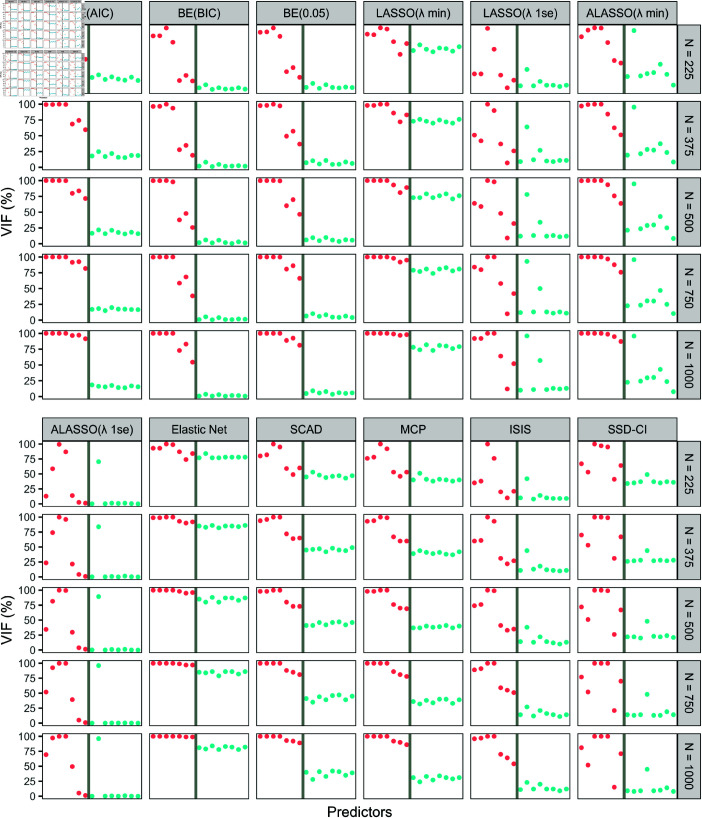
Variable inclusion frequency (VIF) of the twelve variable selection methods with all simulated predictors. 1000 simulations were used to plot and evaluate the variable selection performance of twelve variable selection methods with sample sizes (*N* = 225, *N* = 375, *N* = 500, *N* = 750 and *N* = 1000). The x-axis represents the true predictors $X_{1},\ldots ,X_{15}$. The predictors on to the left of the grey colour vertical solid line are true predictors ($X_{1},\ldots ,X_{7})$ and are represented in red dots while the false predictors on to the right are visualised in blue colour dots $(X_{8},\ldots ,X_{15}$). Variable selection methods: BE(AIC), BE(BIC), BE(0.05), LASSO($\lambda_{min}$), LASSO($\lambda_{1se}$), ALASSO($\lambda_{min}$), ALASSO($\lambda_{1se}$), Elastic Net, SCAD, MCP, ISIS and SSD-CI.

While Fig 1 illustrates variable selection performance based on all predictors, Fig 2 highlights the likelihood of each predictor being selected as significant based on VIF measures in percentage. The reason for the poor performance of Elastic Net and LASSO($\lambda_{min}$) in differentiating true from false predictors is revealed in Fig 2, as each predictor has been selected as significant in at least half of the simulations by recording a VIF above 50%. BE(AIC) was the only method which was able to clearly identify all true predictors with an EPV on or beyond 50% across all sample sizes, indicating it is more appropriate to identify the true predictors.

The individual behavior of predictors, as shown in Fig 2, further highlights the performance of the candidate methods in selecting true predictors, particularly *X*_1_, *X*_6_, and *X*_7_, which have smaller coefficients, with *X*_7_ being the smallest. Additionally, the effect of multicollinearity on the methods’ ability to identify true predictors was also examined.

All methods showcased a difficulty in recognizing the true predictors $X_{5}\;-\;X_{7}$ (except for SSD–CI for *X*_5_), especially at *N* = 225, although this issue waned as the sample size increased. However, ALASSO($\lambda_{1se}$) continued to struggle with *X*_6_ and *X*_7_ and LASSO$(\lambda_{1se}$) with *X*_6_ showing low VIFs (at or below 10%). SSD-CI also had difficulty in identifying *X*_6_, and this issue worsened as sample size increased. Furthermore, SSD-CI, ISIS, LASSO($\lambda_{1se}$) and ALASSO$(\lambda_{1se}$) had trouble identifying *X*_1_ and *X*_2_, the two true predictors with the largest correlations (Fig B in S1 Appendix) despite *X*_1_ having the third smallest coefficient. This issue, however, diminished with the increment of the sample size except for *X*_2_ under SSD-CI as the VIF remained constantly at 50%, irrespective of the sample size.

Regarding false predictors, BE(BIC) followed by BE(0.05) performed best in shrinking VIFs by recording VIFs below 10% and 12% for all false predictors, respectively. However, ALASSO$\left(\lambda_{1se}\right)$ was the overall best in omitting false predictors, recording almost zero VIF for all except *X*_9_. Interestingly, *X*_9_ was selected as significant in nearly all simulations, with a VIF of 70% at *N* = 225, possibly due to its correlation with *X*_6_, a truly significant predictor. BE(AIC) which performed best in terms of identifying true predictors, however, recorded a VIF consistently at 25% (half of that of the lower limit for true predictors) making it inferior compared to the two other versions of BE method and ALASSO$\left(\lambda_{1se}\right)$ in identifying false predictors. This indicates that following identification of true predictors based on VIFs computed under BE(AIC), the dropped predictors need to be further investigated for the insignificance using ALASSO$\left(\lambda_{1se}\right)$ and BE(BIC) methods. However, from *N* = 750 with EPV of 50, the true state of a predictor is possible to be clearly determined just by referring the VIFs generated by the BE(0.05) method. The true predictors record a VIF of 65% while a false predictor barely recorded a VIF beyond 10% (VIF of *X*_9_ was 10.2%).

It was also noted that the rate of false predictor selection was similar within the false predictors and also between SCAD and MCP methods. This rate of false selection also dropped in a similar fashion in SCAD and MCP, unaffected by correlations (Fig B in S1 Appendix). Furthermore, as false predictor VIFs declined, the true predictor VIFs increased parallely by recording 100% for $X_{1}\;-\;X_{4}\text{since}N=750$ and 75% or above for $X_{5}\;-\;X_{7}$. These two methods also performed better than ISIS and comparably to BE(AIC) in identifying the true predictors although overall inferior in avoiding redundant predictors from getting selected to the final model. Similarly to SCAD and MCP, ALASSO($\lambda_{min}$) improved in identifying true predictors, recording a lower Type II error rate (FNR) than SCAD and MCP. Its Type I error rate (FPR) was also lower than these methods, yet its performance of selecting the true model containing only the true predictors as the output remains well below SCAD and MCP due to the persistent false selection of *X*_9_, which correlated with true predictor *X*_3_ (Fig B in S1 Appendix).

LASSO($\lambda_{1se}$)’s variable selection was similarly affected by the correlation structure (Fig B in S1 Appendix). The frequent selection of true predictor *X*_3_ led to false predictor *X*_9_ being selected as significant (VIF of almost 100% at *N* = 1000). Moreover, LASSO($\lambda_{1se})$ falsely selected *X*_11_ instead of *X*_6_, with *X*_11_ being selected in more than 50% of simulations at *N* = 1000, while *X*_6_ was selected in just over 10% of the simulations. SSD-CI exhibited similar behavior, with *X*_11_’s VIF remaining around 50% while the VIF of *X*_6_ dropped together with other false predictors.

In SSD-CI, the failure to identify *X*_6_ affected the average number of predictors selected as the output across the simulations. On average, SSD-CI began by selecting 8.185 predictors and dropped to 6.302 at *N* = 1000 (Table 1), while other methods selected more predictors as the sample size increased. ALASSO($\lambda_{1se}$) consistently selected fewer than the true number of predictors, while Elastic Net and LASSO($\lambda_{min}$) always selected more, nearly double the true number. ISIS, BE(BIC), BE(0.05) and LASSO($\lambda_{1se}$) slightly understated the true number of predictors in the small sample case but matched or slightly exceeded the true number at *N* = 1000. Oppsingly, BE(AIC) almost matched the true number of predictors in the small sample case (7.154 at *N* = 225) and slightly overstated as the sample size increased selecting 8.13 predictors on average as significant at *N* = 1000.

**Table 1:** The average number of predictors selected as significant by each method under each *N.*

Method	Average number of predictors selected as significant				
	N=225	N=375	N=500	N=750	N=1000
ALASSO$\left(\lambda_{1se}\right)$	3.493	4.072	4.426	4.864	5.203
LASSO$\left(\lambda_{1se}\right)$	3.631	5.058	5.944	6.898	7.342
ISIS	4.090	5.235	5.932	6.749	6.910
BE(BIC)	4.438	4.925	5.301	5.810	6.230
BE(0.05)	5.151	5.945	6.275	6.839	7.101
BE(AIC)	7.154	7.515	7.782	8.044	8.130
Contrast	8.185	7.529	7.158	6.700	6.302
ALASSO$\left(\lambda_{min}\right)$	8.269	8.560	9.078	9.458	9.579
MCP	8.282	8.951	9.203	9.364	9.071
SCAD	8.982	9.576	9.729	9.897	9.727
LASSO$\left(\lambda_{min}\right)$	11.201	12.211	12.61	13.178	13.155
Elastic Net	12.564	13.488	13.655	13.674	13.455

**Fig 3:**
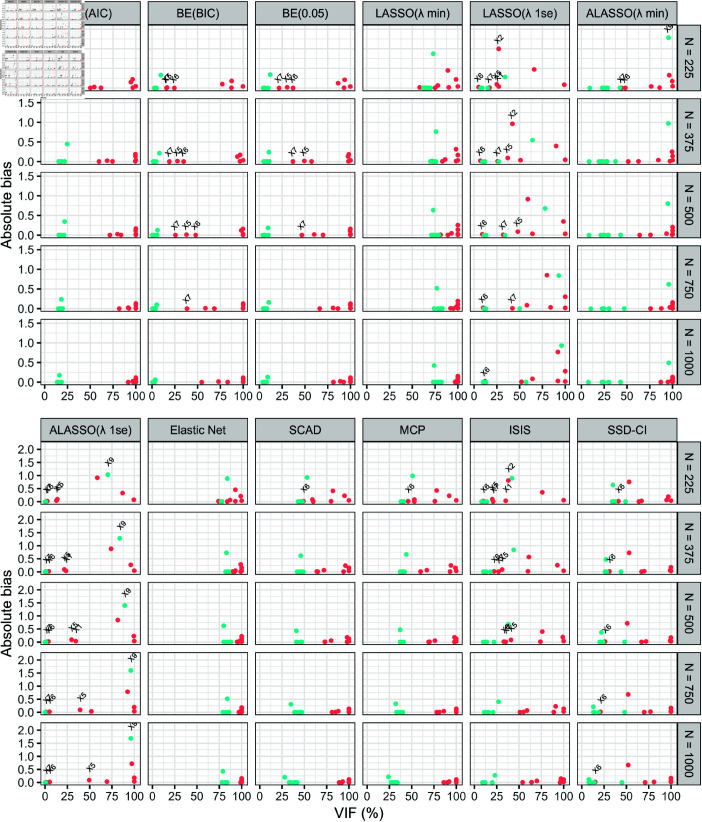
Pairwise performance of each simulated predictor on variable selection and making valid inference using twelve variable selection methods based on variable inclusion frequency (VIF) and absolute bias. The appropriateness of each variable selection method on descriptive modeling was evaluated based on the values computed for each predictor collectively on the percentage of VIF and absolute bias across five sample sizes (*N* = 225, *N* = 375, *N* = 500, *N* = 750 and *N* = 1000). The true predictors ($X_{1},\ldots ,X_{7}$) are denoted in red colour while the false predictors in blue colour. The true predictors with a VIF (%) below 50% and any predictor with an absolute bias beyond 0.5 are also labelled on each graph. Variable selection methods: BE(AIC), BE(BIC), BE(0.05), LASSO($\lambda_{min}$), LASSO($\lambda_{1se}$), ALASSO($\lambda_{min}$), ALASSO($\lambda_{1se}$), Elastic Net, SCAD, MCP, ISIS and SSD-CI.

**Fig 4:**
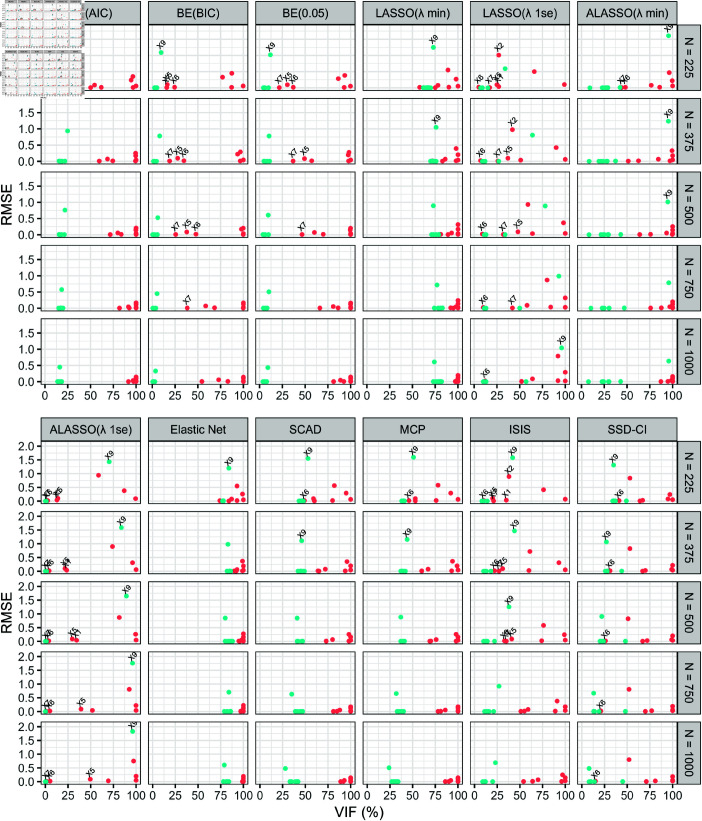
Pairwise performance of each simulated predictor on variable selection and making valid inference were assessed using variable inclusion frequency (VIF) and RMSE computed by twelve variable selection methods. The appropriateness of each variable selection method on descriptive modeling was evaluated based on the values computed for each predictor collectively on the percentage of VIF and RMSE across five sample sizes (*N* = 225, *N* = 375, *N* = 500, *N* = 750 and *N* = 1000). The predictors in red colour represent true predictors while the false predictors are given in blue colour. The true predictors with a VIF (%) below 50% and any predictor with a RMSE greater than 1 are also labelled on each graph. Variable selection methods: BE(AIC), BE(BIC), BE(0.05), LASSO($\lambda_{min}$), LASSO($\lambda_{1se}$), ALASSO($\lambda_{min}$), ALASSO($\lambda_{1se}$), Elastic Net, SCAD, MCP, ISIS and SSD-CI.

Following the variable selection assessment, the properties of the regression coefficient estimates were evaluated using absolute bias and RMSE in Fig 3 and Fig 4, respectively. Since variable selection and inference are equally important in descriptive modeling, the joint behavior of each summary measure with VIF was observed rather than evaluating them in isolation. According to these figures, the methods that positioned true predictors closer to the bottom right corner (large VIF with low absolute bias and RMSE) and false predictors towards the bottom left (low VIF, absolute bias and RMSE) are considered optimal for both variable selection and inference and thereby for descriptive modeling.

Across all methods, absolute bias and RMSE behaved similarly against VIF, with RMSE generally being higher than absolute bias, except in a few cases for ALASSO($\lambda_{1se}$). Under low to moderate sample sizes (N∈[225,500]), BE(AIC) performed best in separating true and false predictors based on three summary statistics, collectively. However, for one false predictor (*X*_9_), the absolute bias was just above 0.5 with RMSE beyond 1 at *N* = 225. Since then, BE(0.05) emerged as the overall best method for both variable selection and inference.

SCAD, MCP and ISIS also showed improvement by shifting true and false predictors into opposite corners of the figures. In addition, it was noted that under all methods, the false predictor against which both the largest measure for absolute bias and RMSE was recorded was *X*_9_. Of note, although *X*_9_ was not labelled under any plot corresponding to BE(BIC) and BE(0.05) in Fig 3, the blue dot with largest summary measure represented *X*_9_. Surprisingly, even though under SSD-CI method, *X*_11_ was most likely to be selected most often among the false predictors, the larger measures for absolute bias and RMSE was recorded for *X*_9_. Between the two screening-based methods, SSD-CI outperformed ISIS in capturing true predictors and estimating regression coefficients more accurately in samples smaller than 500. Furthermore, despite some methods struggling to recognize certain true predictors with a VIF below 50% as superimposed on Figs 3 and 4, both absolute bias and RMSE remained near zero, except for *X*_2_ under LASSO$(\lambda_{1se}$) and ISIS methods, which recorded high measures for both statistics (both at *N* = 225 and LASSO$\left(\lambda_{1se}\right)$ at *N* = 375). However, this issue pertained in LASSO($\lambda_{1se}$), even after *X*_2_ selected with higher probability in subsequent sample sizes. As a result, SSD-CI and ISIS are preferable to LASSO($\lambda_{1se}$) for descriptive modeling.

### 5.2 Performance evaluation based on the red wine dataset

Tables 2 and 3 present the stability measures (VIF, RCB in percentage and RMSDR) computed for each predictor in the red wine dataset across different methods. The dataset’s EPV was 159.9 (1599/10), approximately 160. Based on simulation results, BE (0.05) performed best for descriptive modeling, consistently selecting significant predictors with a VIF of at least 80% at *N* = 1000 (EPV = 66.7).

**Table 2:** Stability measures (VIF, RCB (%) and RMSDR) for predictors in the red wine dataset with a VIF of 100% under the BE(0.05) method.

	Residual sugar			Total sulfur dioxide			Alcohol		
**Method**	**VIF (%)**	**RCB (%)**	**RMSDR**	**VIF (%)**	**RCB (%)**	**RMSDR**	**VIF (%)**	**RCB (%)**	**RMSDR**
BE(0.05)	100	0.964	0.986	100	0.153	1.075	100	0.020	1.145
BE(AIC)	100	0.806	0.983	100	-0.132	1.064	100	0.092	1.141
BE(BIC)	100	2.012	1.054	100	0.304	1.097	100	-0.161	1.205
LASSO($\lambda_{min}$)	100	-1.957	1.040	100	-1.831	1.045	100	-1.403	1.249
LASSO($\lambda_{1se}$)	100	-33.991	4.843	100	-32.570	2.806	100	-20.910	5.244
ALASSO($\lambda_{min}$)	69.0	-65.263	10.709	0.0	NA	7.876	69.4	-22.938	13.535
ALASSO($\lambda_{1se}$)	28.4	-79.848	12.777	0.0	NA	7.876	65.5	-41.638	16.296
Elastic Net	100	-1.547	0.998	100	-2.273	1.057	100	-1.205	1.224
SCAD	100	0.441	1.023	100	0.570	1.043	100	-0.199	1.213
MCP	100	0.442	1.022	100	0.633	1.047	100	-0.210	1.215
ISIS	0.0	NA	13.886	0.0	NA	7.876	0.0	NA	24.263
SSD-CI	0.0	NA	13.886	0.0	NA	7.876	62.8	-10.412	15.068
	**Density**			**Chlorides**			**Fixed acidity**		
**Method**	**VIF (%)**	**RCB (%)**	**RMSDR**	**VIF (%)**	**RCB (%)**	**RMSDR**	**VIF (%)**	**RCB (%)**	**RMSDR**
BE(0.05)	100	0.545	1.149	100	0.885	1.251	100	0.779	1.443
BE(AIC)	100	0.414	1.136	100	0.692	1.246	100	0.497	1.385
BE(BIC)	100	0.856	1.194	100	1.186	1.290	100	1.392	1.550
LASSO($\lambda_{min}$)	100	-1.429	1.234	100	-0.149	1.199	100	-0.816	1.343
LASSO($\lambda_{1se}$)	100	-24.183	6.883	100	-4.705	1.244	100	-12.269	5.249
ALASSO($\lambda_{min})$	100	-59.279	21.873	69.4	14.818	5.163	69.4	-15.832	22.179
ALASSO($\lambda_{1se})$	99.9	-66.385	21.181	69.4	20.616	5.273	69.4	-25.445	23.570
Elastic Net	100	-1.648	1.232	100	0.334	1.146	100	-0.937	1.376
SCAD	100	0.307	1.195	100	0.414	1.224	100	0.383	1.402
MCP	100	0.303	1.197	100	0.464	1.225	100	0.398	1.405
ISIS	0.0	NA	27.806	0.0	NA	9.464	100	-43.733	18.161
SSD-CI	100	-48.053	14.627	0.1	10.010	9.459	100.0	-24.768	10.737

**Table 3:** Stability measures (VIF, RCB (%) and RMSDR) for predictors in the red wine dataset with a VIF below 100% under the BE(0.05) method.

	Free sulfur dioxide			Sulphates			Citric acid			Volatile acidity		
**Method**	**VIF (%)**	**RCB (%)**	**RMSDR**	**VIF (%)**	**RCB (%)**	**RMSDR**	**VIF (%)**	**RCB (%)**	**RMSDR**	**VIF (%)**	**RCB (%)**	**RMSDR**
BE(0.05)	99.5	0.010	1.147	92.9	1.969	1.595	25.1	92.209	1.440	22.7	125.707	1.343
BE(AIC)	100	-0.261	1.133	97.4	0.489	1.557	41.6	71.726	1.375	36.5	89.408	1.325
BE(BIC)	94.6	0.014	1.174	86.9	6.765	1.796	13.3	120.218	1.479	9.4	174.273	1.348
LASSO($\lambda_{min}$)	100	-3.844	1.154	99.9	-4.303	1.450	92.0	15.079	1.125	92.2	3.325	1.188
LASSO($\lambda_{1se}$)	87.0	-56.771	3.481	89.6	-41.461	2.732	98.4	74.533	1.432	58.9	6.104	0.921
ALASSO($\lambda_{min}$)	0.0	NA	5.538	40.2	-51.820	4.392	69.4	202.476	2.680	8.5	-12.561	1.030
ALASSO($\lambda_{1se}$)	0.0	NA	5.538	16.5	-59.348	4.835	67.5	204.200	2.733	3.8	-6.341	1.041
Elastic Net	100	-3.210	1.139	99.9	-4.081	1.475	96.2	15.821	1.202	94.2	7.829	1.212
SCAD	100	-0.112	1.137	98.8	-0.032	1.510	52.0	56.355	1.295	62.0	54.461	1.282
MCP	100	-0.051	1.139	97.8	0.774	1.512	49.2	58.836	1.300	55.0	68.038	1.290
ISIS	0.0	NA	5.538	0.0	NA	5.168	0.0	NA	1.365	0.0	NA	1.062
SSD-CI	0.0	NA	5.538	0.0	NA	5.168	100.0	408.666	5.830	34.3	-218.015	1.649

Six predictors were identified as significant in every bootstrap sample, each recording a VIF of 100% (Table 2). Among them, *fixed acidity* was the only predictor consistently selected as important across all methods, except for ALASSO, which recorded a VIF of 69.4%. *Density* was the second most important predictor, achieving a VIF of 100% across all methods except ISIS (VIF = 0%). BE(0.05) further refined the selection of significant predictors, identifying *residual sugar*, *total sulfur dioxide*, *alcohol*, *density*, *chlorides* and *fixed acidity* as important, each with a VIF of 100% and an RCB below 1% (Table 2). This conclusion was supported by other BE approaches, LASSO, Elastic Net, SCAD, and MCP, all of which recorded a VIF of 100% for these six predictors.

Although *free sulfur dioxide* (Table 3), was not among the primary six predictors, it was selected as important in nearly every resample, recording a VIF of 99.5% and an RCB close to zero, suggesting its significance. It was noted that the seven predictors identified as significant under BE(0.05) recorded RMSDR values above 1, except for *residual sugar*, which remained below 1.5. Additionally, *sulphates* recorded a VIF of 92.9% making it a potential predictor,though its RCB and RMSDR slightly exceeded 1.5. Conversely, BE(0.05) selected *citric acid* and *volatile acidity* in only about 25% of the resamples (25.1% and 22.7%, respectively) (Table 3), with significantly high RCBs (92.209% and 125.707%, respectively), suggesting they were likely insignificant.

To further verify the insignificance of *citric acid* and *volatile acidity*(as identified by BE(0.05)), we examined them using using ALASSO($\lambda_{1se}$) and BE(BIC). ALASSO($\lambda_{1se}$) recorded a VIF of 3.8% for *volatile acidity*,while BE(BIC) reported 9.4%, confirming its insignificance. Similarly, BE(BIC) recorded a VIF of 13.3% for *citric acid*,reinforcing BE(0.05)’s conclusion. However, ALASSO($\lambda_{1se}$) contradicted this by assigning a VIF of 67.5% to *citric acid*, incorrectly labeling it as important, similar to *X*_9_ in the simulation study. These findings further justified the weakness of Elastic Net and LASSO($\lambda_{min}$) in distinguishing true predictors from false ones, as both methods selected all predictors with VIFs of at least 92%.

From an inferential perspective, we assessed the stability of coefficient measures using percentage RCB and RMSDR. For the eight predictors deemed significant by BE(0.05), RCB values remained below 2%, supporting their inclusion in the final model. However, all significant predictors recorded RMSDR values above 1, indicating that variable selection increased the variability of the estimated regression coefficients. Other BE approaches, as well as MCP and SCAD, supported this conclusion by producing similar stability measure values (except for the RCB of 6.765% (Table 3) for *sulphates* under the BE(BIC) method). In contrast, *citric acid* and *volatile acidity* exhibited RCB values above 90%, reinforcing their insignificance in explaining pH levels in red wine. This conclusion was further supported by other BE approaches, which recorded RCBs exceeding 70% for these two predictors.

RCB values were not computed for certain predictors under ALASSO, ISIS, and SSD-CI methods, where all these approaches did not select *total sulfur dioxide* and *free sulfur dioxide* in any resample. Consequently, these predictors recorded a VIF of 0% and ‘NA’ for RCB. Notably, these were the only predictors never selected under either ALASSO approach. Among these methods, the simulation results suggested that ISIS performed well in large sample sizes with high EPVs. However, in this dataset, ISIS was the weakest method for selecting true predictors, identifying only *fixed acidity* as significant (VIF = 100%) (Table 2) while assigning a VIF of 0% to all other predictors.

SSD-CI performed better than ISIS in identifying potentially important predictors, selecting *fixed acidity* and *density* (both VIF = 100%) and *alcohol* (VIF = 62.8%). However, it also erroneously selected *citric acid* (VIF = 100%), contradicting BE(0.05). The high RCB (408.7%) and RMSDR (5.83) for *citric acid* suggested its irrelevance for inference purposes. Similarly, both ALASSO approaches selected *citric acid* in nearly 70% of resamples,but with RCB values exceeding 200% and RMSDR above 2.5, further confirming its insignificance (Table 3).

For model stability, Table 4 presents the most frequently selected model across subsamples for each method. *Fixed acidity*, *density* and *alcohol* were consistently included in all models, except for ISIS. Similar to the VIF results, the most frequently selected model under BE(0.05) also included *residual sugar*, *chlorides*, *free sulfur dioxide*, *total sulfur dioxide* and *sulphates* (in 51.7% subsamples). BE(0.05) and BE(BIC) exhibited similar predictor selection patterns, suggesting comparable performance. Consistent with the simulation studies, Elastic Net and LASSO($\lambda_{min}$) were the weakest in model stability, as their most frequently selected model contained all predictors. Surprisingly, LASSO($\lambda_{1se}$), SCAD and MCP, which performed well in large sample sizes, also included all predictors in their most frequently selected models. ISIS further demonstrated poor performance compared to stability measures, selecting only *fixed acidity* in its most common model, which was the sole model chosen across all 1000 resamples.

**Table 4:** Differing models obtained by applying each candidate method on 1000 subsamples.

Method	Different models	Most frequently selected model	
		Variables included	sub_MSF(%)
BE(0.05)	10	Fixed acidity, Residual sugar, Chlorides, Free sulfur dioxide, Total sulfur dioxide, Density, Sulphates, Alcohol	51.7
BE(AIC)	06	Fixed acidity, Citric acid, Residual sugar, Chlorides, Free sulfur dioxide, Total sulfur dioxide, Density, Sulphates, Alcohol	38.4
BE(BIC)	11	Fixed acidity, Residual sugar, Chlorides, Free sulfur dioxide, Total sulfur dioxide, Density, Sulphates, Alcohol	70.6
LASSO($\lambda_{min}$)	05	All predictors	84.4
LASSO($\lambda_{1se}$)	11	All predictors	44.8
ALASSO($\lambda_{min}$)	06	Fixed acidity, Citric acid, Residual sugar, Chlorides, Density, Sulphates, Alcohol	35.9
ALASSO($\lambda_{1se}$)	14	Fixed acidity, Citric acid, Chlorides, Density, Alcohol	31.2
SCAD	07	All predictors	31.9
MCP	08	All predictors	29.7
SSD-CI	05	Fixed acidity, Citric acid, Density, Alcohol	43.6
ISIS	01	Fixed acidity	100
Elastic Net	05	All predictors	90.4

## 6 Discussion

We conducted a comprehensive simulation study to compare the variable selection and inference properties of several widely used statistical methods in observational studies for linear regression modeling. Additionally, we included a CI-based statistical approach inspired by a SSD-based method to evaluate its performance in variable selection under observational settings.

This simulation study examined how well the methods selected predictors under scenarios where strong and weak associations pertained among true predictors, false predictors and between true and false predictors. We also evaluated the ability of these methods to identify true predictors with both large and small coefficients and their capacity to make valid inferences. The performance of each method was assessed under varying sample sizes (EPVs).

Elastic net and LASSO($\lambda_{min}$) [12] performed best in identifying the true predictors but at the cost of including a significant number of false positives (on average, 5 out of 8 false predictors). Other studies [12, 25, 60] support this finding, but the selection of both true and false predictors with VIFs above 0.7 suggests these methods are less suitable for variable selection in low-dimensional data. However, according to [66], these methods outperformed BE, SCAD, and ALASSO methods in risk prediction, especially in datasets with few events. Interestingly, BE (BIC) consistently excelled in excluding false predictors, while BE(AIC) and ALASSO($\lambda_{min}$) performed better in capturing true predictors.

The inverse relationship between FPR and FNR made identification of method(s) which perform overall best in variable selection more challenging. VIF measure was therefore, more productive with this regards and further facilitated in identifying the strengths and weaknesses of each methods in detail. Based on the VIF measures (Fig 2), none of the methods successfully identified all true predictors while eliminating redundant ones. However, examining the figures suggests that selecting true predictors using BE(AIC) with a VIF cut-off value of 50%. or higher could be beneficial. The results of our study, however, illustrates the importance of justifying the insignificance of the predictors dropped by BE(AIC) method using the VIF measures computed for the corresponding dropped predictors under ALASSO($\lambda_{1se}$) method, followed by BE(BIC). In addition, BE(0.05) method was effective in selecting true predictors, in datasets with an events-per-variable (EPV) ratio beyond 50.

The error rates in Fig 1 further revealed a similar behaviour in variable selection between LASSO($\lambda_{1se}$) and SSD-CI method. While improvements in one error rate sometimes came at the cost of worsening another, the overall variable selection performance remained comparable. The VIF figures further supported this similarity. The error rates and stability measures confirmed that ISIS outperformed LASSO($\lambda_{1se}$ in descriptive modeling.

It was also noted that multicollinearity and the magnitude of the coefficients notably affected the performance of most methods. ALASSO($\lambda_{1se})$ excelled at eliminating redundant predictors with near–zero VIF but was hindered by the inclusion of predictor *X*_9_ in most simulations. The moderate association between *X*_9_ and true predictor *X*_3_ led to *X*_9_ being selected as significant, while true predictors *X*_1_ and *X*_6_ were omitted. Below an EPV of 50, all methods struggled to identify the true status of at least one of true predictors $X_{5}-X_{7}$, with BE(AIC) being least affected. *X*_7_ had the smallest coefficient, followed by *X*_6_, and the relationship between *X*_5_ with *X*_6_ seemed to influence the selection of *X*_5_ as significant. Not only performed poorly in selection of true predictors with low coefficients, LASSO($\lambda_{1se}$), ALASSO($\lambda_{min}$), ALASSO($\lambda_{1se}$) and SSD-CI methods underperformed in both variable selection and handling multicollinearity, regardless of sample size.

Our findings align with [85], who also reported the poor performance of LASSO in the presence of multicollinearity. While Elastic Net is generally considered to perform better in such cases [25], our study did not support this. Contrarily, [62], found that LASSO and ALASSO methods outperformed in multicollinear settings with ALASSO($\lambda_{1se}$) showing superior variable selection compared to LASSO. In [60] the authors observed that LASSO and Elastic Net performed well in identifying true predictors and eliminating false ones in a study on serum biomarkers of overweight and obesity, but we did not replicate this finding. Furthermore, [74] noted that LASSO tends to select only one of the highly correlated factors, while our study found that LASSO often generated more complex models by including both true predictors and non-predictors.

The poor performance of penalized regression methods and the SSD-CI method made BE(AIC) or BE(0.05) the overall best performers across the sample sizes considered. In line with study [11], BE was deemed most appropriate for variable selection in low-dimensional settings [23, 86, 87]. In a simulation study comparing LASSO and BE, BE produced a more parsimonious model. Additionally, [88] demonstrated that BE(AIC), when applied to bootstrap data, yielded the most parsimonious model. BE also improved model selection in datasets with higher EPV and fewer missing values compared to LASSO. In the study [5], which focused on variable selection for prediction, BE(AIC) and BE(0.05) had yielded better parsimony in small data sets compared to tree-based methods. This is in line with our findings. However, BE(BIC) yielded sparser models but understating the true number of predictors.

Although BE(AIC) and BE(0.05) performed the best overall in selecting true predictors while omitting false predictors reasonably well within the study range, SCAD and MCP show potential to surpass these methods under very large EPVs. Within the study range, these two methods already performed comparable to BE (AIC) in selecting true predictors, maintaining an FNR similar to BE(AIC) and even lower than BE(0.05). The false predictor selection rate of the BE approaches remained unaffected by sample size, while the FPR showed a continuous decline. This ongoing improvement in eliminating false predictors suggests that SCAD and MCP may outperform classical methods in very large sample sizes.

Since this simulation study focused on assessing the variable selection performance of the methods for descriptive modeling, we further evaluated these methods based on the validity of the inferences made on the coefficients. Here, we demonstrated the importance of observing the performance of each method using absolute bias and RMSE paired with VIF, instead of looking at them separately. Surprisingly, even though *X*_9_ was not frequently selected by the BE methods, higher absolute bias and RMSE values were recorded for this predictor. This was the only concern raised regarding inference in BE approaches, as the absolute bias for all true predictors remained below 0.25. Since, *X*_9_ was the only predictor against which BE(AIC) recorded an absolute bias beyond 0.5 and RMSE beyond 1 at *N* = 225 and that too be a false predictor, BE(AIC) remained to be overall best with respect to both variable selection and inference perspective. Based on pairwise performance, BE(0.05) became highly favorable to apply on datasets with EPV greater than 50 for descriptive modeling.

The main concern with respect to other methods was the production of biased estimates for the coefficients of the true predictors. Among them, LASSO($\lambda_{1se}$) performed the worst in terms of inference, followed by ALASSO($\lambda_{1se}$) and SSD-CI. As the sample size increased, the absolute bias and RMSE of the remaining methods decreased, with the greatest improvement observed for SCAD and MCP followed by ISIS. These results suggest that these methods may be better suited for both variable selection and inference in very large sample sizes. Additionally, Figs 3 and 4 highlight that the SSD-CI method outperformed LASSO($\lambda_{1se}$) and generally provided better inference than ISIS, particularly in datasets with small sample sizes and an EPV at or below 25.

In real-world application, all methods identified *fixed acidity* and *density* (except ISIS) as potentially significant factos. Given the EPV of around 160, our simulation suggests that BE(0.05) is the preferred method for selecting significant predictors, and according to this approach, *residual sugar*, *total sulfur dioxide*, *alcohol*, *chlorides*, *free sulfur dioxide* and *sulphates* were also selected. This selection is further justified by the two other BE approaches, MCP and SCAD methods. The stability measures sub_MSF corresponding to BE(0.05) and BE(BIC) support these selections as the most frequently selected model across the subsamples consist of these eight predictors. The ALASSO($\lambda_{1se}$) and BE(BIC) together confirmed the insignificance of *volatile acidity* dropped by BE(0.05), except for *citric acid*, where background knowledge may assist in determining its significance.

Surprisingly, although simulations suggested that the ISIS method would excel in descriptive modeling with very large sample sizes, it performed the worst on the observed dataset. It selected only *fixed acidity* and failed to identify *density* as an important predictor, despite *density* having the largest average bootstrap coefficient across all candidate predictors in other methods (Table A in S1 Appendix). While the sure screening property theoretically guarantees that ISIS retains important predictors as *N* approaches infinity [89], these findings cast doubt on its real-world applicability. Furthermore, although simulations indicated that ISIS outperforms SSD-CI in identifying true predictors under large sample sizes, real data analysis suggested otherwise. Several limitations were encountered in this study. These findings were only tested in linear regression models with only one correlation structure, primarily low to moderate, between the predictors. Also, the type of predictors involved in this study was limited to numeric form. Additionally, our comparison focused on variable selection methods commonly used in observational studies, which some may view as a limitation. Future research could explore modern methods, such as Bayesian model averaging and tree-based methods. Moreover, the real-world dataset used in this study did not perfectly mirror the simulated data, as it contained high correlations between numerous predictors. Despite being a prominent dataset in model comparison studies for variable selection, the high correlation among input factors made it less practical for comparing candidate methods.

## 7 Conclusion

In summary, this paper compares the variable selection performance of several widely used statistical methods and a novel approach inspired by the SSD-based factor screening method, within the framework of descriptive modeling. We recommend evaluating variable selection performance using VIF measures, rather than relying solely on error rates. Additionally, for descriptive modeling, method recommendations should consider the combined performance of a summary measure used to assess inference quality and the VIF measures computed for the predictors. Our simulation study further highlights that, under certain conditions, BE methods may outperform modern variable selection methods in both variable selection and inference, despite common critiques of stepwise regression. Additionally, The SSD-CI method showed performance comparable to LASSO($\lambda_{1se}$) as a screening tool and was superior for inference in small samples, outperforming both LASSO($\lambda_{1se}$) and ISIS. These findings suggest that SSD-based screening methods warrant further exploration for variable selection in observational studies. In conclusion, no single method is optimal across all scenarios, and practitioners should approach variable selection in observational studies with caution.

## Supporting information

S1 Appendix Fig A. Distribution of the predictors designed for the simulation study. The histogram of each design predictor (X_1_,…,X_15_) was plotted by simulating a dataset of 50,000 sampling units according to the study design.
Fig B. Correlogram revealing the correlations between the predictors of the simulation study.
The empirical correlation coefficients of the simulated predictors were plotted using a dataset of 50,000 sampling units simulated following the study design.
Fig C. Correlation plot corresponding to the predictors in the real life dataset.
The correlogram reveals the correlation coefficients computed for the thirteen predictors in the red wine dataset.
Table A. Distribution of coefficient estimates computed for the predictors in the red wine dataset.
The mean (standard deviation) of the distribution of bootstrap coefficient estimates computed for each predictor under each candidate statistical method.
Table B. R code for the SSD-CI method.
Requires R to open; download R software package from https://cran.r-project.org/ if necessary.
(DOCX)
