# Supplementary material for: Variable selection methods for descriptive modeling
Source: PLoS One. 2025 Jun 2;20(6):e0321601. doi: 10.1371/journal.pone.0321601 (PMC12129470; doi:10.1371/journal.pone.0321601)
Supplement: S1 Appendix — The histogram of each design predictor (X1,…,X15) was plotted by simulating a dataset of 50,000 sampling units according to the study design. Fig B. Correlogram revealing the correlations between the predictors of the simulation study. The empirical correlation coefficients of the simulated predictors were plotted using a dataset of 50,000 sampling units simulated following the study design. Fig C. Correlation plot corresponding to the predictors in the real life dataset. The correlogram reveals the correlation coefficients computed for the thirteen predictors in the red wine dataset. Table A. Distribution of coefficient estimates computed for the predictors in the red wine dataset. The mean (standard deviation) of the distribution of bootstrap coefficient estimates computed for each predictor under each candidate statistical method. Table B. R code for the SSD-CI method. Requires R to open; download R software package from https://cran.r-project.org/ if necessary. (DOCX) [file pone.0321601.s001.docx]

Appendix for

**Variable selection methods for descriptive modeling**

A. D. V. Tharkeshi T. Dharmaratne^1^, Alysha De Livera^2^, Stelios Georgiou^1^, Stella Stylianou^1, *^

^1^ School of Science, RMIT University, Melbourne, Australia

^2^ Engineering and Mathematical Sciences, La Trobe University, Bundoora, Australia

^*^ Corresponding author. E-mail:  [stella.stylianou@rmit.edu.au](mailto:haoyt@mail.sysu.edu.cn)


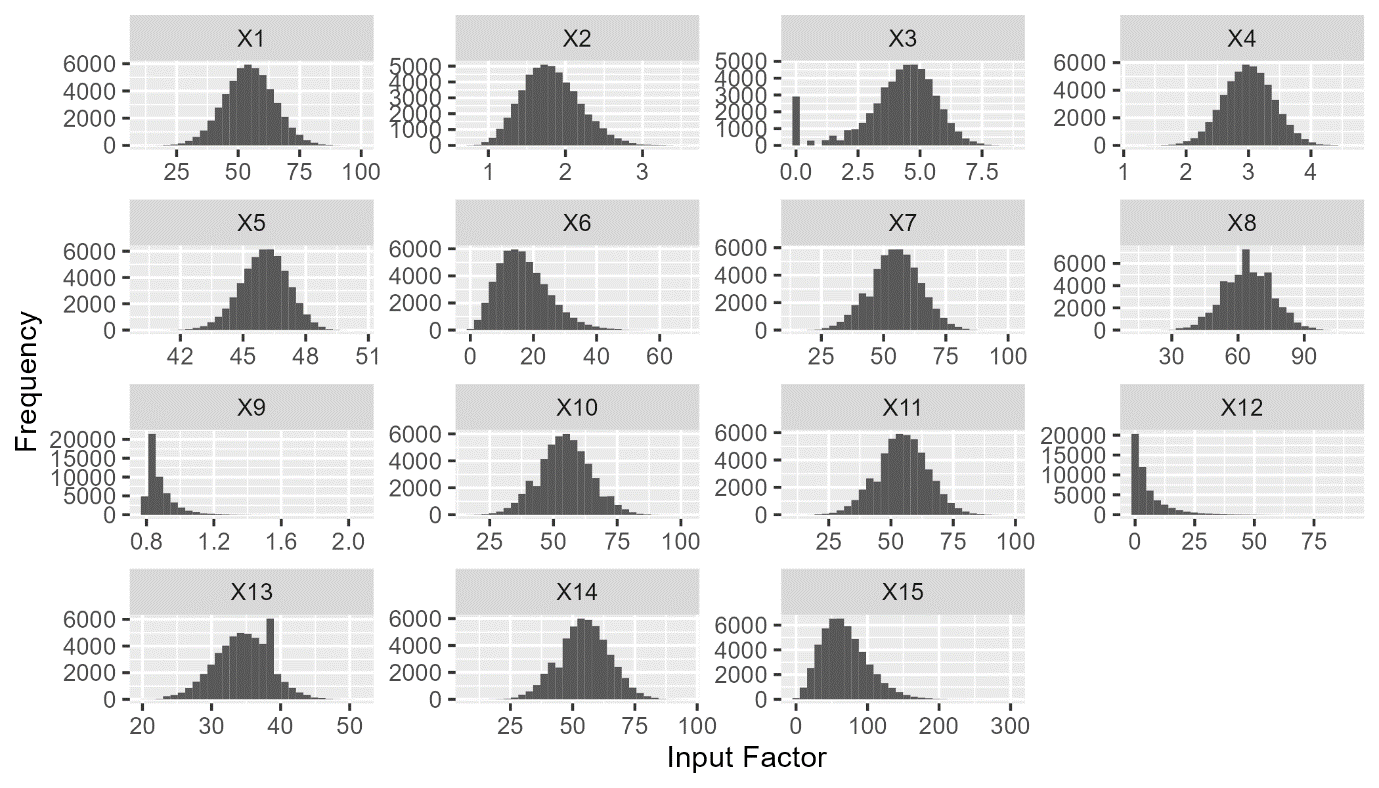
**Fig A. Distribution of the predictors designed for the simulation study.** The histogram of each design predictor $\left( X_{1}, \ldots, X_{15} \right)$was plotted by simulating a dataset of 50,000 sampling units according to the study design.


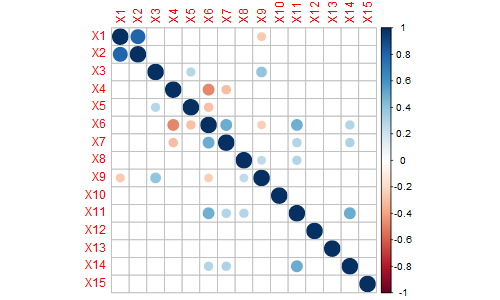
**Fig B. Distribution of the predictors designed for the simulation study.** The empirical correlation coefficients of the simulated predictors were plotted using a dataset of 50,000 sampling units simulated following the study design.


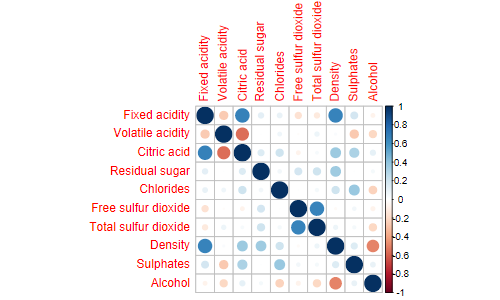
**Fig C. Distribution of the predictors in the real data .** Correlation plot corresponding to the predictors in the real-life dataset.

**Table A. Distribution of coefficient estimates computed for the predictors in the red wine dataset.** The mean (standard deviation) of the distribution of bootstrap coefficient estimates computed for each predictor under each candidate statistical method.

| Method | Intercept | Fixed acidity | Volatile acidity | Citric acid | Residual sugar | Chlorides | Free sulfur dioxide | Total sulfur dioxide | Density | Sulphates | Alcohol |
| --- | --- | --- | --- | --- | --- | --- | --- | --- | --- | --- | --- |
| BE(AIC) | -61.1  (2.62) | -0.098  (0.003) | 0.016  (0.021) | -0.023  (0.026) | -0.026  (0.002) | -0.509  (0.067) | 0.002  (0.0003) | -0.001  (0.0001) | 64.9  (2.63) | -0.076  (0.023) | 0.072  (0.003) |
| BE(BIC) | -61.4  (2.71) | -0.099  (0.003) | 0.010  (0.020) | -0.016  (0.027) | -0.026  (0.002) | -0.512  (0.069) | 0.002  (0.0003) | -0.001  (0.0001) | 65.2  (2.72) | -0.077  (0.027) | 0.072  (0.004) |
| BE(0.05) | -61.2  ( 2.64) | -0.098  (0.003) | 0.014  (0.021) | -0.021  (0.027) | -0.026  (0.002) | -0.510  (0.067) | 0.002  (0.0003) | -0.001  (0.0001) | 65.0  (2.65) | -0.077  (0.024) | 0.072  (0.003) |
| LASSO($\lambda_{min}$) | -59.9  (2.71) | -0.097  (0.003) | 0.016  (0.019) | -0.028  (0.022) | -0.025  (0.002) | -0.505  (0.064) | 0.002  (0.0003) | -0.001  (0.0001) | 63.7  (2.72) | -0.073  (0.021) | 0.071  (0.004) |
| LASSO($\lambda_{1se}$) | -45.3  (3.40 ) | -0.085  (0.003) | 0.011  (0.013) | -0.045  (0.020) | -0.017  (0.002) | -0.482  (0.062) | 0.001  (0.0003) | -0.0005  (0.0001) | 49.0  (3.41) | -0.043  (0.023) | 0.057  (0.004) |
| ALASSO($\lambda_{min}$) | -22.8  (33.2) | -0.060  (0.037) | 0.002  (0.006) | -0.058  (0.041) | -0.006  (0.005) | -0.423  (0.263) | 0.000  (0.000) | 0.000  (0.000) | 26.3  (33.4) | -0.016  (0.024) | 0.040  (0.025) |
| ALASSO($\lambda_{1se}$) | -18.1  (24.0) | -0.053  (0.034) | 0.001  (0.005) | -0.057  (0.043) | -0.002  (0.003) | -0.445  (0.275) | 0.000  (0.000) | 0.000 (0.000) | 21.7  (24.2 ) | -0.007  (0.016) | 0.029  (0.023) |
| Elastic Net | -59.8  (2.65) | -0.096  (0.003) | 0.017  (0.019) | -0.029  (0.023) | -0.025  (0.002) | -0.507  0.061) | 0.002  (0.0003) | -0.001  (0.0001) | 63.5  (2.66) | -0.074  (0.022) | 0.071  (0.004) |
| SCAD | 61.1  (2.76) | -0.098  (0.003) | 0.015  (0.020) | -0.023  (0.025) | -0.026  (0.002) | -0.508  (0.065) | 0.002  (0.0003) | -0.001  (0.0001) | 64.8  (2.77) | -0.076  (0.022) | 0.072  (0.004) |
| MCP | -61.1  (2.76) | -0.098  (0.003) | 0.015  (0.020) | -0.023  (0.025) | -0.026  (0.002) | -0.508  (0.065) | 0.002  (0.0003) | -0.001  (0.0001) | 64.8  (2.78) | -0.076  (0.023) | 0.072  (0.004) |
| ISIS | 3.77  (0.048) | -0.055  (0.006) | 0.000 (0.000) | 0.000 (0.000) | 0.000 (0.000) | 0.000 (0.000) | 0.000 (0.000) | 0.000 (0.000) | 0.000 (0.000) | 0.000 (0.000) | 0.000 (0.000) |
| SSD-CI | -29.9  (14.000) | -0.073  (0.008) | -0.005  (0.014) | -0.134  (0.033) | 0.000 (0.000) | -0.001  (0.018) | 0.000 (0.000) | 0.000 (0.000) | 33.6  (13.8) | 0.000 (0.000) | 0.040  (0.031) |

**Table B. R code for the SSD-CI method.** Requires R to open; download R software package from <https://cran.r-project.org/> if necessary.

| Line NO. | R codes and comments |
| --- | --- |
| 1  2  3  4  5  6  7  8  9  10  11  12  13  14  15  16  17  18  19  20  21  22  23  24  25  26  27  28  29  30  31  32  33  34  35  36  37  38  39  40  41  42  43  44  45  46  47  48  49  50  51  52  53  54  55  56  57  58  59  60  61  62  63  64  65  66  67  68  69  70 | # Main function  # Input parameter: (X, Y) for data; a: Number of predictors incorporated to construct a confidence interval (75% of X in this study); alpha: Significance level (0.05 in this study)  ## X: Predictors  ## Y: Response  SSD_CI=function(X,Y,a,alpha){  #Stage 1: Standardize X and Y  X<-scale(X,T,T)  ##Total factors  m<-ncol(X)  ##Convert X to a matrix  X1<-data.matrix(X, rownames.force = NA)  Y<-scale(Y)  #Stage 2: Compute contrasts  ##Transpose of X  Tr_X<-t(X1)  contrasts<-Tr_X%*%Y  ##Get the absolute values  abs_contrasts<-abs(contrasts)  ##Order the absolute contrasts  Order_contrasts_tab<- abs_contrasts[order((abs_contrasts[,1]),decreasing=FALSE),1,drop=FALSE]  order_contrasts_all<-as.numeric(Order_contrasts_tab)  names(order_contrasts_all)<-row.names(Order_contrasts_tab)  #Stage 3: Perform the iterative process  i<-0  Variance<-Inf  ##Perform variable selection while m-i>P and Var(i)<Var(i-1)    while((m-i)>a){  ##Drop the variable with largest contrast, in each iteration  Order_contrasts<-order_contrasts_all[1:(m-i)]  ##length of the selected contrasts  x<-length(Order_contrasts)  ##Select “a” number of contrasts with highest absolute values  active<-Order_contrasts[(x-(a-1)):x]    ##variance and standard deviation of the selected “a” values  Var<-var(active)  SD<-sd(active)  ## Construct confidence interval  ###Degrees of freedom in t distribution  df<-x-1  tscore<-qt(alpha/2, df, lower.tail=FALSE)  crit_region<-tscore*SD    #Check for stopping criterion  if(Var>Variance\|crit_region>max(Order_contrasts)){break}    else{  Variance<-Var  UCL<-max(Order_contrasts)-crit_region  LCL<-(-max(Order_contrasts))+crit_region    #Step 4: Select and store the potential significant predictors  var_selection <-row.names(contrasts)[which(contrasts<LCL\|contrasts>UCL)]  }  i<-i+1    }  return(var_selection)  }  # The end |
